# Radiopaque tissue transfer and X-ray system *versus* standard specimen radiography for intraoperative margin assessment in breast-conserving surgery: randomized clinical trial

**DOI:** 10.1093/bjsopen/zrac091

**Published:** 2022-08-10

**Authors:** Angrit Stachs, Julia Bollmann, Annett Martin, Johannes Stubert, Toralf Reimer, Bernd Gerber, Steffi Hartmann

**Affiliations:** Multidisciplinary Breast Unit, Department of Radiology, University of Rostock, Rostock, Germany; Multidisciplinary Breast Unit, Department of Gynaecology and Obstetrics, University of Rostock, Rostock, Germany; Multidisciplinary Breast Unit, Department of Gynaecology and Obstetrics, University of Rostock, Rostock, Germany; Multidisciplinary Breast Unit, Department of Gynaecology and Obstetrics, University of Rostock, Rostock, Germany; Multidisciplinary Breast Unit, Department of Gynaecology and Obstetrics, University of Rostock, Rostock, Germany; Multidisciplinary Breast Unit, Department of Gynaecology and Obstetrics, University of Rostock, Rostock, Germany; Multidisciplinary Breast Unit, Department of Gynaecology and Obstetrics, University of Rostock, Rostock, Germany

## Abstract

**Background:**

Reduction of positive margin rate (PMR) in breast-conserving surgery (BCS) of non-palpable breast cancer remains a challenge. The efficacy of intraoperative specimen radiography (SR) is unclear. This randomized trial evaluated whether the PMR was reduced by the use of devices that allow precise localization of the affected margins.

**Methods:**

Patients with microcalcification-associated breast cancer undergoing planned BCS were enrolled. Study participants were randomized to receive either SR with radiopaque tissue transfer and X-ray system (KliniTray^TM^) or the institutional standard procedure (ISO). In all patients with a radiological margin less than 5 mm, an immediate re-excision was conducted. The primary outcome was the PMR. Risk factors for positive margins and the effect of immediate re-excision on final surgery were secondary analyses.

**Results:**

Among 122 randomized patients, 5 patients were excluded due to the extent of primary surgery and 117 were available for analysis. Final histopathology revealed a PMR of 31.7 per cent for the KliniTray^TM^ group and 26.3 per cent for the ISO group (*P* = 0.127). Independent factors for positive margins were histological tumour size more than 30 mm (adjusted OR (aOR) 10.73; 95 per cent c.i. 3.14 to 36.75; *P* < 0.001) and specimen size more than 50 mm (aOR 6.65; 95 per cent c.i. 2.00 to 22.08; *P* = 0.002). Immediate re-excision due to positive SR led to an absolute risk reduction in positive margins of 13.6 per cent (from 42.7 to 29.1 per cent).

**Conclusion:**

Specimen orientation with a radiopaque tissue transfer and X-ray system did not decrease the PMR in patients with microcalcification-associated breast cancer; however, SR and immediate re-excision proved to be helpful in the reduction of PMR.

**Registration number:**

DRKS00011527 (https://www.drks.de).

## Teaser

This prospective randomized study aimed to answer the question of whether in patients with breast-conserving surgery (BCS) of ductal carcinoma *in situ* (DCIS), the need for a second surgery due to affected margins can be reduced with a radiopaque tissue transfer and X-ray system (KliniTray^TM^) for intraoperative specimen radiography.

No significant difference was found between specimen radiography with the KliniTray^TM^ system *versus* institutional standard orientation; however, specimen radiography followed by immediate re-excision led in 32 per cent of cases to conversion from initially positive to finally negative pathological margins in BCS of predominantly intraductal breast cancer associated with microcalcifications.

Based on these findings, specimen radiography is of limited use to reduce the positive margin rate in BCS of DCIS independently of the technique for specimen orientation.

## Introduction

During the last decade, screening programmes have led to an increased incidence of noninvasive and early-stage breast cancer^1,2^. For these, breast-conserving therapy is standard in surgical therapy with equivalent or even better survival outcomes compared with mastectomy^3–5^. Thereby, a negative margin status is the most important factor to prevent local recurrence^6,7^.

For ductal carcinoma *in situ* (DCIS), a negative margin width of 2 mm is generally recommended^8,9^. The percentage of positive margins in DCIS varies between 18 and 63 per cent^10–13–14^. If tumour-free margins are not achieved by initial surgery, further secondary surgeries as two-stage procedures are necessary. Additional surgery, whether as re-excision or secondary mastectomy, submits the patient to an increased treatment burden, including the risk of anaesthesia and poor cosmetic results. That is why intraoperative margin assessment is of crucial importance; however, frozen section assessment is not recommended for non-palpable breast lesions as well as microcalcifications^15–17^.

Specimen radiography (SR) is widely used not only to document the excision of the targeted lesion but also to provide information about margin involvement. Nevertheless, the role of SR in decreasing the positive margin rate (PMR) is still unclear. Some studies could demonstrate a reduction of secondary surgery rate by targeted intraoperative re-excision^18,19^, whereas other studies failed to show an effect on PMR^13,17^. A recent review of the performance of SR in DCIS revealed a wide range of sensitivity from 22 to 77 per cent and moderate specificity from 52 to 100 per cent. The authors noted a high risk of bias in the index test (SR) predominantly being retrospectively assessed. Moreover, only a poor correlation (48–56 per cent) between the direction of the shortest distance measured with SR compared with that of final pathology was reported^10^. This may be explained by errors in orientation during SR as intraoperative SR requires optimal and fast cooperation between the surgeon, radiologist, and pathologist. Attempts to improve SR results have been made using digital breast tomosynthesis^20^ or remote intraoperative techniques^19^.

Devices allowing an exact topographic localization of the lesion in the resected tissue could reduce re-excision rates via intraoperative detection and exact localization of involved margins, as has recently been shown for breast-conserving surgery (BCS) after neoadjuvant chemotherapy by Jamaris *et al.*^21^. The KliniTray™ system (KLINIKA Medical, Usingen, Germany) is a transport and X-ray system for tissue samples to determine the free resection margins. This device enables a reliable anatomical assignment by the use of X-ray opaque orientation markers. In the case of positive radiological margins, a targeted immediate re-excision to the corresponding localization can be performed. This aim of this study was to determine whether the use of a radiopaque tissue transfer system (KliniTray™) improved the accuracy and efficiency of immediate re-excision in the case of positive radiological margins in comparison with institutional standard orientation (ISO).

## Methods

The KliniTray™ trial was designed as a prospective, open-label RCT comparing SR with a radiopaque tissue transfer system (KliniTray™) or ISO for BCS of predominantly intraductal carcinoma of the breast, with a 1:1 allocation ratio. The results are reported following the CONSORT guidelines.

The trial was approved by the University of Rostock Institutional Review Board (protocol A2016-0149) and registered in the German Register of Clinical Studies (DRKS) as DRKS-ID DRKS00011527.

### Participants

After obtaining informed consent from patients, patients were enrolled in this single-centre study from 1 January 2017 to 15 October 2020. Patients were eligible if the following inclusion criteria were fulfilled: non-palpable lesion with microcalcification on mammography; complete preoperative imaging (mammography, ultrasound, magnetic resonance imaging optional); histologically confirmed DCIS or invasive breast cancer with predominant noninvasive components; planned BCS with intraoperative SR; and age 18 years or older. Patients with primarily planned mastectomy as well as palpable lesions were excluded. Mammographic size estimation was based on digital mammograms with spot magnification. All patients underwent localization with standard guidewire by one of two experienced breast radiologists. BCS was carried out by one of five senior breast surgeons.

### Randomization

Randomization was undertaken on a 1:1 basis by permutated block randomization to intraoperative SR by the use of a radiopaque tissue transfer system (intervention) or by ISO (control). Patients were randomized by way of the closed envelope technique. There were an equal number of envelopes containing KliniTray™ and control group assignments provided by an independent statistician (Günther Kundt). Each patient received a random ID number that was used at all phases of the study. After patient consent was obtained, the envelope was opened by one of the breast surgeons.

### KliniTray™ and ISO procedure

**Fig. 1:**
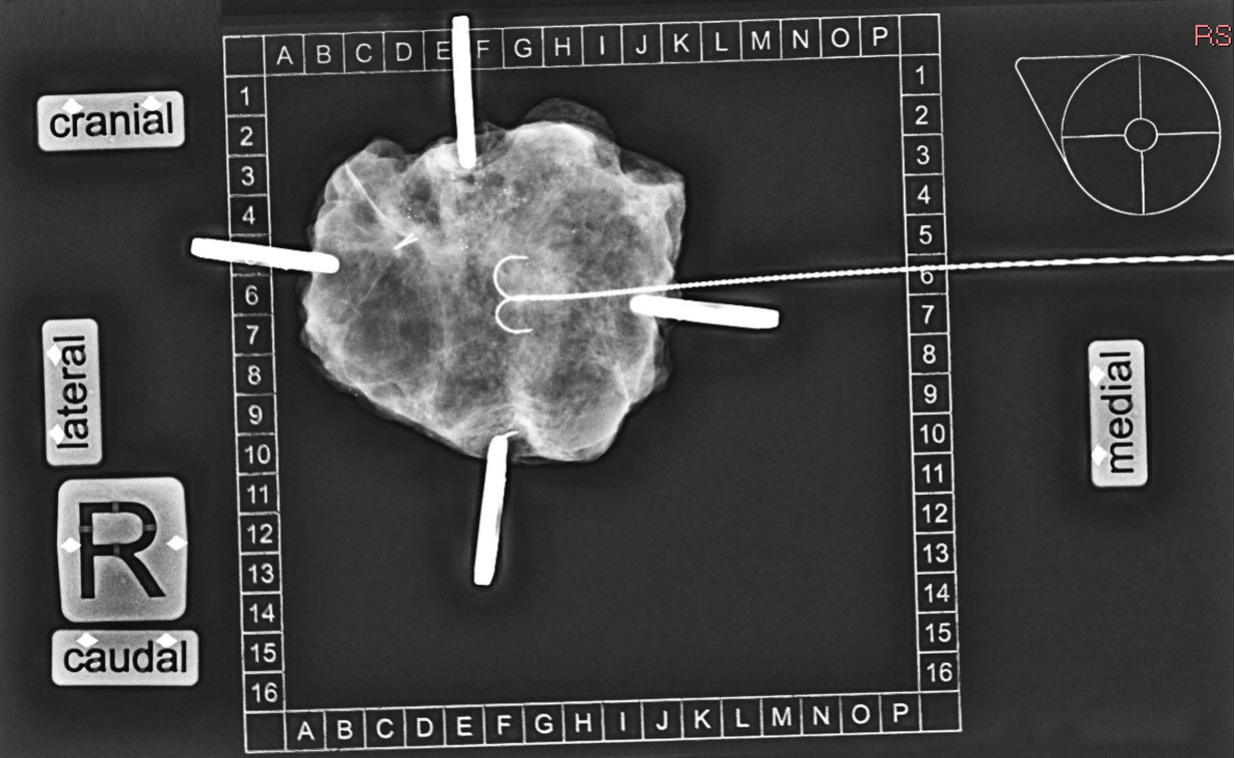
Specimen radiography with the KliniTray™ system after lumpectomy

For SR performed in the ISO (control) group, suture threads were applied to the breast specimen in the operating room: one thread for the areolar margin, two threads for the peripheral margin, and three threads for the superficial or cutaneous margin. For the patients in the intervention group, surgical specimens were fixed on a tissue transfer and X-ray system with radiopaque topographic markers (KliniTray™) (*Fig. 1*). In all cases, specimens were sent to the Breast Imaging Service for SR using a digital mammography unit (Hologic Dimensions; Hologic Deutschland, Wiesbaden-Nordenstadt, Germany) and appropriate compression. One of two experienced breast radiologists immediately reported the presence/absence of the lesion and the width and direction of the closest margin. The radiologist's margin assessment was carried out without the knowledge of the histopathological findings according to the study protocol, immediate re-excision of the appropriate margin was performed if the radiological margin was less than 5 mm. Histopathology of the first surgical specimen was defined as the initial specimen. If intraoperative re-excision was performed, results of initial surgery plus re-excision were defined as final histopathology. Initial and final findings were identical in cases where no re-excision was perfomed.

The wire is located in the central part of the specimen with microcalcifications reaching the cranial margin.

### Outcomes

The primary outcome was the PMR using final histopathology as the standard. According to international guidelines, a positive/involved pathological margin was defined as a resection margin less than 2 mm in case of pure DCIS, and as a resection margin less than 1 mm if an invasive tumour was found^8^. Concerning SR, radiological margins less than 5 mm were defined as positive. Secondary analyses were the evaluation of factors associated with PMRs as well as analysing the effect of immediate re-excision on PMR.

### Statistical analysis

The sample size was calculated with study planning software nQuery7.0 (STATCON, Witzenhausen, Germany). A reduced incidence of PMR in the KliniTray™ group was postulated. Based on previously published results^22^, it was calculated that 55 patients would be needed in each group to provide 80 per cent power to detect a reduction of 50 per cent (52 *versus* 26 per cent) in the PMR at a two-sided *α* level of 0.05. A total of 122 patients (61 per group) was aimed for to correct for an estimated 10 per cent drop-out rate. *t* tests for continuous variables and *χ*² tests for categorical variables regarding differences between groups were performed. The primary outcome was analysed with cross-tables with *χ*² testing and binary logistic regression to adjust for baseline covariables.

## Results

### Patient and tumour characteristics

**Fig. 2:**
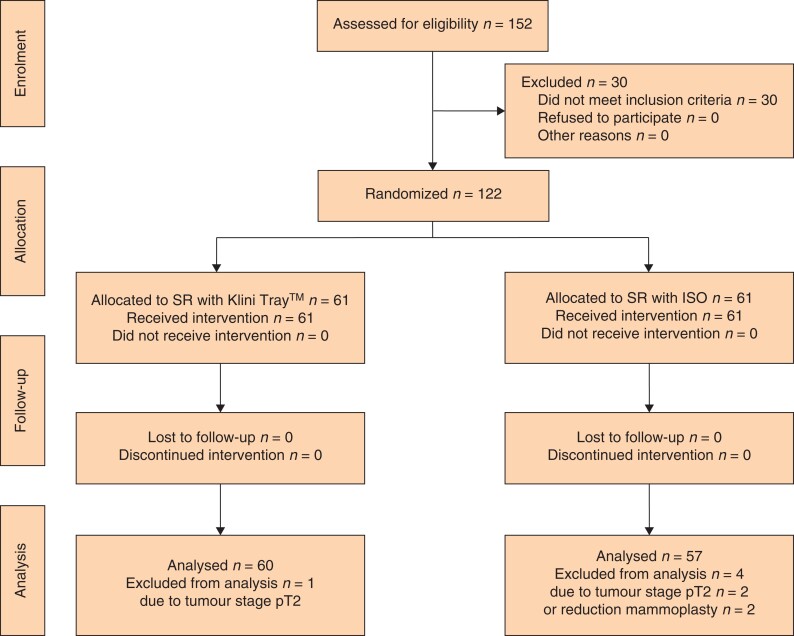
Study flow diagram

SR, specimen radiography; ISO, institutional standard orientation.

A total of 122 patients met the selection criteria mentioned above. Of them, 61 were randomized in the KliniTray™ group and 61 in the control group. Postoperative invasive tumour category pT2 (three patients) and reduction mammoplasty (two patients) led to exclusion of five patients after randomization, one in the intervention group and four in the control group (*Fig. 2*). Final statistical analysis was performed with 117 patients (KliniTray™ group, 60 patients; control group, 57 patients). Patient characteristics are summarized in *Table 1*. The mean age of the study population was 61.2 years. Pure DCIS was present in 98 patients, microinvasive ductal carcinoma in one patient, and tumour category pT1 in 18 patients. There was no significant difference between the study groups regarding the following variables: tumour stage, grading, preoperative biopsy classification, mammographic tumour size, morphology and distribution of microcalcifications, and multifocality as well as presence or absence of comedo necrosis; however, there were significant differences between the KliniTray™ group and the ISO group concerning the median histopathological DCIS size (30 mm *versus* 25 mm; *P* = 0.025) and median specimen size (55 mm *versus* 50 mm; *P* = 0.035). Moreover, DCIS cases in the KliniTray™ group were significantly more oestrogen receptor-positive in comparison with the control group (90.0 *versus* 75.4 per cent; *P* = 0.032).

**Table 1:** Patient, tumour and radiological characteristics

Characteristic	Total *n* = 117	ISO *n* = 57	KliniTray™ *n* = 60
**Age (years) mean(s.d.)**	61.2(8.41)	62.4(8.98)	60.0(7.89)
**Tumour stage**			
pTis	98 (83.8)	47 (82.5)	51 (85.0)
pT1mic	1 (0.9)	0 (0.0)	1 (1.7)
pT1a	2 (1.7)	0 (0.0)	2 (3.3)
pT1b	6 (5.1)	4 (7.0)	2 (3.3)
pT1c	10 (8.5)	6 (10.5)	4 (6.7)
**Grading**			
G1	6 (5.1)	3 (5.3)	3 (5.0)
G2	47 (40.2)	23 (40.4)	24 (40.0)
G3	64 (54.7)	31 (54.4)	33 (55.0)
**Minimal biopsy classification**			
B5a	100 (85.5)	48 (84.2)	52 (86.7)
B5b	15 (12.8)	9 (15.8)	6 (10.0)
B5c	2 (1.7)	0 (0.0)	2 (3.3)
Median mammographic size (mm), median (range)	17.0 (2.0–58.0)	17.0 (2.0–54.0)	17.0 (4.0–58.0)
**Morphology of microcalcifications**			
Fine linear/branching	12 (10.3)	7 (12.3)	5 (8.3)
Fine pleomorphic	67 (57.3)	29 (50.9)	38 (63.3)
Coarse heterogenous	38 (32.3)	21 (36.8)	17 (28.3)
**Distribution of microcalcifications**			
Grouped	57 (48.7)	26 (45.6)	31 (51.7)
Linear/segmental	60 (51.3)	31 (54.4)	29 (48.3)
**Multifocality**			
No	104 (88.9)	49 (86.0)	55 (91.7)
Yes	13 (11.1)	8 (14.0)	5 (8.3)
**Comedo necrosis**			
No	17 (14.5)	8 (14.0)	9 (15.0)
Yes	100 (85.5)	49 (86.0)	51 (85.0)
**Oestrogen receptor status**			
Positive	97 (82.9)	43 (75.4)	54 (90.0)
Negative	20 (17.1)	14 (24.6)	6 (10.0)
Median DCIS size (mm), median (range)	25.0 (2.0–100)	25.0 (2.0–64.0)	30.0 (10.0–100)
Median specimen size (mm), median (range)	56.9 (30–120)	50.0 (30–90)	55.0 (30–120)
**Histopathology of initial specimen**			
R0	67 (57.3)	32 (56.1)	35 (58.3)
R1	50 (42.7%)	25 (43.9)	25 (41.7)
**Histopathology of final specimen (immediate re-excision included)**			
R0	83 (70.9)	42 (73.7)	41 (68.3)
R1	34 (29.1)	15 (26.3)	19 (31.7)
**Specimen radiography (number of involved margins)**			
0	73 (62.4)	32 (56.1)	41 (68.3)
1	22 (18.8)	15 (26.3)	7 (11.7)
>1	22 (18.8)	10 (17.5)	12 (20.0)
**Intraoperative re-excision**			
No	76 (65.0)	36 (63.2)	40 (66.7)
Yes	41 (35.0)	21 (36.8)	20 (33.3)
**Two-stage (second) surgery**			
No	85 (72.6)	42 (73.7)	43 (71.7)
Re-excision	19 (18.2)	10 (17.5)	9 (15.0)
Mastectomy	13 (11.1)	5 (8.8)	8 (13.3)

Values are n (%) unless otherwise indicated. ISO, institutional standard orientation; DCIS, ductal carcinoma *in situ*.

### Primary outcome (results of surgical treatment)

After BCS, initial histopathology revealed positive margins in 50 of 117 (42.7 per cent) patients. There was no difference in initial PMR between the study groups. In 44 (37.6 per cent) patients, intraoperative SR revealed involved margins. According to the study protocol, an immediate intraoperative re-excision was executed in 41 patients, 20 in the KliniTray™ group and 21 in the control group. In three patients, immediate re-excision was not possible due to unfavourable breast size. Final PMR was 29.1 per cent and revealed no significant difference between KliniTray™ group and control group (31.7 *versus* 26.3 per cent; *P* = 0.330) (*Table 1*). Two-stage (second) surgery was carried out in 28.3 per cent of patients in the KliniTray™ group as well as 26.3 per cent in the ISO group and included re-excision in 19 and secondary mastectomy in 13 patients.

### Factors associated with positive margins on the initial specimen and after immediate re-excision

Due to observed imbalance between the study groups concerning histopathological tumour size and specimen size, but not with mammographic tumour size, defined variables with impact on histopathology were analysed (*Table 2*). A positive correlation was found between initial PMR and mammographic tumour size more than 20 mm (*P* = 0.029), histological tumour size more than 30 mm (*P* < 0.001), positive SR (*P* = 0.003), and radiological underestimation more than 10 mm (*P* = 0.021), whereas estrogen receptor (ER) status, specimen size and orientation (KliniTray™ *versus* standard) did not influence initial PMR significantly. After immediate re-excision, an increased risk for positive margins in final histopathology was observed for patients with histopathological DCIS size more than 30 mm (unadjusted OR 4.81; 95 per cent c.i. 2.05 to 11.3; *P* < 0.001) and for specimen size more than 50 mm (adjusted OR (aOR) 2.32; 95 per cent c.i. 1.02 to 5.27; *P* = 0.044). Specimen orientation (KliniTray™ *versus* ISO) did not affect the final PMR. Nevertheless, specimen orientation was included in the multivariate logistic regression model to analyse the independence of the influencing factors (considering that histological tumour size and specimen size were not well balanced between the study groups). Independent factors associated with positive margins on final specimen were histological tumour size (aOR 10.73; 95 per cent c.i. 3.14 to 36.75; *P* < 0.001) and specimen size (aOR 6.65; 95 per cent c.i. 2.0 to 22.08; *P* = 0.002) (*Table 3*).

**Table 2:** Positive margin rate (PMR) on initial and final specimen

	Total	Initial PMR	*P*	Final PMR	*P*
**Mammographic DCIS size (mm)** ^*^			0.029		0.397
≤20	69 (59.0)	24 (34.8)		18 (26.1)	
>20	48 (41.0)	26 (54.2)		16 (33.3)	
**Histological DCIS size (mm)** ^*^			<0.001		<0.001
≤30	78 (66.7)	24 (30.8)		14 (17.9)	
>30	39 (33.3)	26 (66.7)		20 (51.3)	
**Specimen radiography**			0.003		0.220
Negative (≥5)	53 (45.3)	15 (28.3)		12 (22.6)	
Positive (<5)	64 (54.7)	35 (54.7)		22 (34.4)	
**Specimen size (mm)**			0.160		0.044
≥50	75 (64.1)	29 (38.7)		17 (22.7)	
<50	42 (35.9)	21 (50.0)		17 (40.5)	
**Oestrogen receptor status**					0.522
Positive	97 (82.9)	38 (39.2)	0.072	27 (27.8)	
Negative	20 (17.1)	12 (50.0)		7 (35.0)	
**Study group**			0.853		0.524
ISO	57 (48.7)	25 (43.9)		15 (26.3)	
KliniTray™	60 (51.3)	25 (41.7)		19 (31.7)	

* Pure DCIS or predominantly DCIS among pT1 invasive breast carcinoma. ISO, institutional standard orientation; DCIS, ductal carcinoma *in situ*.

Values are n (%), as there is no otherwise indicated values.

**Table 3:** Univariate and multivariate logistic regression model for predicting the risk of finally positive margins

	Univariate logistic regression			Multivariate logistic regression		
	uOR	95 c.i.	*P*	aOR	95 c.i.	*P*
**Mammographic DCIS size (mm)** >20 *versus* ≤20*	1.42	0.63–3.17	0.397	1.05	0.48–2.94	0.719
**Histological DCIS size** >30 *versus* ≤30*	4.81	2.05–11.3	<0.001	10.73	3.14–36.75	<0.001
**Specimen size (mm)** <50 *versus* ≥50*	2.32	1.02–5.27	0.044	6.65	2.00–22.08	0.002
**Oestrogen receptor** Negative *versus* Positive*	1.40	0.50–3.87	0.522			
**Specimen orientation** KliniTray™ *versus* ISO*	1.30	0.58–2.90	0.524	1.18	0.48–2.94	0.719

* Reference. aOR, adjusted OR; ISO, institutional standard orientation; DCIS, ductal carcinoma *in situ*; uOR, unadjusted OR.

### Comparison of specimen radiography with initial histopathological results

To verify the diagnostic accuracy of the SR, mammographs were reviewed by an independent radiologist blinded to the results of histopathology. In 35 of 50 (70 per cent) patients with initially involved margins (on histopathology), SR indicated positive margins, whereas in 15 patients SR revealed negative margins (false-negative rate of 30 per cent). Additionally, radiologists reported positive margins using SR in 29 of 67 (43.3 per cent) patients with histopathologically negative margins. Sensitivities, specificity, positive predictive value, negative predictive value, and accuracy for the total study population and both study groups alone are shown in *Table 4*. SR with KliniTray™ tended to be most accurate, although differences were not statistically significant.

**Table 4:** Diagnostic performance of specimen radiography with regard to specimen orientation

		Total (*n* = 117)		KliniTray™ (*n* = 60)		ISO (*n* = 57)	
	Margins	SR negative	SR positive	SR negative	SR positive	SR negative	SR positive
**Initial histology**	Free margins (*n* = 67)	38	29	21	14	17	15
	Involved margins (*n* = 50)	15	35	6	19	9	16
Sensitivity (95% c.i.)		70.0 (55.4–82.1)		76.0 (54.9–90.6)		64.0 (42.5–82.0)	
Specificity (95% c.i.)		56.7 (44.0–68.8)		60.0 (42.1–76.1)		53.1 (34.7–70.9)	
PPV (95% c.i.)		54.7 (46.5–62.6)		57.6 (46.1–68.3)		51.6 (40.0–63.1)	
NPV (95% c.i.)		71.7 (61.2–80.25)		77.8 (62.4–88.1)		65.4 (50.5–77.8)	
Accuracy (95% c.i.)		62.4 (53.0–71.2)		66.7 (53.3–78.3)		57.9 (44.1–70.9)	

ISO, institutional standard orientation; SR, specimen radiography; PPV, positive predictive value; NPV, negative predictive value.

### Effects of immediate re-excision on final histopathology results

The results of the initial surgery in comparison to final histopathology are presented in *Table 5*. Due to positive margins on SR, immediate re-excision was carried out in 41 patients and led to a statistically significant conversion from initially positive margins to finally negative margins in 16 out of 50 (32 per cent) patients (*P* < 0.001, McNemar). An absolute risk reduction of 13.6 per cent was observed from 42.7 per cent (initial PMR) to 29.1 per cent (final PMR). Of 50 patients with positive margins on initial pathology, 32 had immediate re-excision and 18 did not. Of the 32 patients, 16 had finally free margins. The risk reduction is thus 50 per cent, and the number needed to treat (NNT) is two, meaning that two patients have to undergo immediate re-excision to convert one patient from initially positive to finally negative margins.

**Table 5:** Comparison of initially and finally positive margins with regard to specimen orientation

		Final histology					
		Total (*n* = 117)		KliniTray™ (*n* = 60)		ISO (*n* = 57)	
	Margins	Not involved	Involved	Not involved	Involved	Not involved	Involved
**Initial histology**	Not involved (*n* = 67)	67	0	35	0	32	0
	Involved (*n* = 50)	16	34	6	19	10	15

ISO, institutional standard orientation.

## Discussion

In the present study, comparisons of the PMR between the randomized study groups KliniTray™ *versus* ISO did not reveal any difference in patients with predominantly intraductal carcinoma of the breast. This is the first prospective randomized trial comparing surgical outcomes of BCS with a radiopaque tissue transfer system *versus* ISO in patients with microcalcification-associated breast lesions. The lack of expected superiority of the KliniTray™ system may have several reasons. First, the PMR for all patients was 29.1 per cent, which is much lower than expected from previous work (PMR of 51.6 per cent)^22^, on which the sample size calculation of the present study was based. Second, there was an imbalance between the study groups regarding median DCIS size, median specimen size, and ER positivity, but not for mammographic size. As tumour size is one of the main factors associated with positive margins and risk for re-excision^23,24^, bias in these study results cannot be excluded. In univariate as well as multivariate analysis, histologic tumour size more than 30 mm and specimen size more than 50 mm increased the risk for positive margins, whereas mammographic size and specimen orientation did not have any impact on PMR; however, stratification for tumour size was not possible, as tumour size is not known before surgery and mammographic size is not sufficiently accurate to estimate tumour size^22,25,26^. Underestimation is more common in low-grade DCIS and non-comedo DCIS, as these are less likely to calcify^27^.

The reported PMR in the present study of 29.1 per cent is comparable to a recent meta-analysis, describing positive margins in 18–63 per cent of BCS for DCIS^10^. Moreover, the risk of re-operation is up to three times higher in patients with noninvasive ductal carcinoma compared with invasive carcinoma^28^. A previous retrospective study from the same breast unit in the present study described a PMR of 51.6 per cent for 92 patients with DCIS treated with BCS^22^. The significant decrease of PMR in the present study is probably related to the prospective study design with a defined standard of immediate re-excision in the case of positive SR. Through the use of immediate re-excision, 32 per cent of cases with initially positive margins shifted to finally uninvolved margins. This is in line with other studies, describing a conversion rate of up to 35 per cent by using selective intraoperative margin assessment^29–31^.

In the present study, the sensitivity of SR was moderate (the probability that SR will be positive if the pathological margin is positive), with a reported false-negative rate of 30 per cent. Additionally, a false-positive rate of 43.3 per cent was observed. The calculation of the NNT revealed that two patients have to be immediately re-excised to prevent one case with finally positive margins; however, unnecessary re-excision can lead to unfavourable cosmetic results and impaired quality of life. The accuracy of SR was 62.4 per cent, which is similar to a recent meta-analysis by Versteegden *et al*., in which accuracy ranged between 55–95 per cent. Interestingly, the accuracy of SR in the KliniTray™ group was remarkably higher than in the standard group (66.7 *versus* 57.9 per cent). The fact that this had no measurable effect on PMR between the two groups may be related to the higher median DCIS size in the KliniTray™ group of 30 mm. In comparison, the median tumour size of 1491 patients with pure DCIS who underwent BCS from 1996 to 2010 at the MD Anderson Cancer Center was 10 mm^32^. The low diagnostic performance of SR may partly be explained by a mammographic underestimation of DCIS size^22^.

Several attempts have been made to prevent positive margins after BCS of breast cancer. An overview of different intraoperative techniques of margin assessment is given in the review by Gray *et al.*^17^. Concerning intraoperative guidance techniques of non-palpable lesions, radioactive seed localization (RSL) seemed to be associated with a lower risk of positive margins in comparison with wire-guided localization (WGL) (RSL *versus* WGL, OR 0.51 95 per cent c.i. 0.36 to 0.72)^33^. Similar results have been found for radioguided occult lesion localization^34^, although the study focused only on cases with invasive breast cancer. A Cochrane analysis revealed no significant differences regarding surgical outcomes between the different localization techniques, and therefore the recommendation for continued use of WGL as a well known technique that allows for flexibility in selected cases with extensive microcalcification was given^35^.

Another widely used method of margin assessment in BCS remains intraoperative digital specimen mammography, with a special mammography device directly in the operation theatre. Studies have shown a reduction in positive margins in addition to a reduction in operating time, but these studies included few pure DCIS^19,36^. A novel approach to finding positive margins is the MarginProbe device with radiofrequency spectroscopy^37,38^. Although the use of MarginProbe was found to reduce positive margins in one randomized trial, it was accompanied by a high false-positive rate^39^. Therefore, the MarginProbe device cannot be recommended for routine use in BCS.

The use of intraoperative pathological techniques has been studied predominantly in invasive breast carcinoma. A retrospective analysis of 688 patients with DCIS from the Mayo Clinic (Rochester, USA), in whom an intraoperative frozen section was performed revealed intraoperatively positive or close margins in 63 per cent of cases. Through immediate re-excision, a PMR of 1.2 per cent was finally achieved^12^. As frozen section analysis of all margins causes additional costs and prolonged time of surgery the question of cost-effectiveness should be addressed. Osborne *et al.* demonstrated that the frozen section is cost-effective if the PMR is otherwise more than 26 per cent^40^. Another retrospective analysis of 588 patients with DCIS undergoing BCS revealed that only macroscopic assessment by pathologists reduced positive margins (OR 0.54, *P* = 0.002), whereas specimen mammography did not have any influence^13^. Unfortunately, all studies did not include details on the radiological appearance of DCIS. The opinion of the authors is that an intraoperative pathological examination is not useful in the case of microcalcifications, as the specimen should be completely reprocessed according to the guidelines^8^. Finally, the question arises whether re-excision is necessary in every case with close margins. Although a minimal margin of 2 mm for DCIS is generally recommended, there exist data from MD Anderson Cancer Center showing that there is no higher risk of local recurrence in BCS of DCIS with margin width less than 2 mm receiving adjuvant radiotherapy^31,32^.

The major limitation of this study is a false-estimated reduction of PMR for the study cohort as the control group also had a lower PMR than assumed. The unequal distribution of tumour size in the two groups may also represent bias; however, a randomized trial with stratification for tumour size is difficult to perform as mammography allows only inaccurate size estimation. Due to the short follow-up, oncological outcomes such as local recurrence rates or survival are not reported for the study cohorts. The present study has several strengths, including that the focus was on a clinically relevant (screening) population including exclusively non-palpable breast lesions associated with microcalcifications. Furthermore, differentiation was made between the initial and final PMR, which allowed a determination of the effect of SR and immediate re-excision as well. Therefore, the prospective study design with a robust number of cases provides evidence of the efficacy of SR for intraductal breast cancer.

The findings of this prospective randomized trial support the limited use of SR for intraoperative margin assessment to reduce the PMR in BCS of breast cancer predominantly associated with microcalcifications; however, there was no evidence of additional benefit with a special radiopaque tissue transfer system.

## Supplementary Material

zrac091_Supplementary_Data

## Data Availability

All data generated and analysed during this study are included in this published article. The presented analyses are based on an anonymous database which means no individual patient data are available. Please contact the corresponding author in case of data request for network analyses.
